# Native Vineyard Non-*Saccharomyces* Yeasts Used for Biological Control of *Botrytis cinerea* in Stored Table Grape

**DOI:** 10.3390/microorganisms9020457

**Published:** 2021-02-22

**Authors:** Antonio Domenico Marsico, Matteo Velenosi, Rocco Perniola, Carlo Bergamini, Scott Sinonin, Vanessa David-Vaizant, Flavia Angela Maria Maggiolini, Alexandre Hervè, Maria Francesca Cardone, Mario Ventura

**Affiliations:** 1 Consiglio per la Ricerca in Agricoltura e L’Analisi dell’Economia Agraria-Centro di Ricerca Viticoltura ed Enologia (CREA-VE), Via Casamassima 148, 70010 Turi, Italy; adomenico.marsico@crea.gov.it (A.D.M.); matteo.velenosi@tiscali.it (M.V.); rocco.perniola@crea.gov.it (R.P.); carlo.bergamini@crea.gov.it (C.B.); 2 Dipartimento di Biologia, Università degli Studi di Bari “Aldo Moro”, Via Orabona 4, 70124 Bari, Italy; flavia.maggiolini@uniba.it; 3 Equipe VAlMiS (Vin, Aliment, Microbiologie, Stress), UMR Procédés Alimentaires et Microbiologiques, AgroSupDijon—Université de Bourgogne/Franche-Comté, IUVV, Rue Claude Ladrey, BP 27877, 21000 Dijon, France; Sinonin.Scott@u-bourgogne.fr (S.S.); vanessa.david@u-bourgogne.fr (V.D.-V.); rvalex@u-bourgogne.fr (A.H.)

**Keywords:** biological control, table grape, *Botrytis cinerea*, yeasts, *Lachancea thermotolerans*, *Metschnikowia pulcherrima*

## Abstract

Postharvest spoilage fungi, such as *Botrytis cinerea*, are considered the main cause of losses of fresh fruit quality and vegetables during storage, distribution, and consumption. The current control strategy is the use of SO_2_ generator pads whose application is now largely under observation. A high quantity of SO_2_ can be deleterious for fresh fruits and vegetables and it is not allowed in organic agriculture. For this reason, great attention has been recently focused on identifying Biological Control Agents (BCA) to implement biological approaches devoid of chemicals. In this direction, we carried out our study in isolating five different non-*Saccharomyces* yeast strains from local vineyards in the South of Italy as possible BCA. We performed both in vitro and in vivo assays in semi-commercial conditions on detached grape berries stored at 0 °C, simulating the temperature normally used during cold storage, and obtained relevant results. We isolated three *M. pulcherrima* strains and one *L. thermotolerans* strain able to largely antagonize the development of the *B. cinerea*, at both in vitro and in vivo conditions. In particular, we detected the ability of the three isolates of *M. pulcherrima* strains Ale4, N20/006, and Pr7 and the *L. thermotolerans* strain N10 to completely inhibit (100% in reduction) the mycelial growth of *B. cinerea* by producing fungistatic compounds. We found, using an extracellular lytic enzymes activity assay, that such activity could be related to lipid hydrolyzation, β-1,3-glucanase and pectinase activity, and pectinase and protease activity, depending on the yeasts used. Results from our in vitro assays allowed us to hypothesize for *M. pulcherrima* strains Ale4 and N20/006 a possible combination of both the production of soluble metabolites and volatile organic compounds to antagonize against *B. cinerea* growth. Moreover, in semi-commercial conditions, the *M. pulcherrima* strain N20/006 and *L. thermotolerans* strain N10 showed relevant antagonistic effect also at low concentrations (with a significantly reduction of ‘slip skin’ incidence of 86.4% and 72.7%, respectively), thus highlighting a peculiar property to use in commercial development for organic agriculture and the handling process.

## 1. Introduction

The Apulia region is the largest producer of table grape in Italy, accounting for 57% of the national production [1], and it is one of the top table grape-growing regions in the world.

Fresh table grape is a very delicate fruit, being subjected to deteriorations during postharvest handling and cold storage, which can result in stem browning, berry drop and microbial rots [2,3,4]. Fungal decay in postharvest is mainly due to the polyphagous and ubiquitous fungus *Botrytis cinerea* Pers.: Fr. (teleomorph: *Botryotinia fuckeliana* (de Bary) Whetzel) that is able to grow and spread even at low temperatures (< −0.5 °C) [5]. This fungus can develop under different conditions such as in the field, during storage, marketing, and even after customer purchase. In stored table grape, without appropriate conditions, symptoms of infections start with small necrosis on the skin, which enlarges in brown spots. In those areas, the fungus produces macerating enzymes below the skin, that separate the cuticle from the flesh (slip skin). Later, on those areas white fungal mycelium starts growing, which originates gray conidia. Wounds or openings in fruit surfaces serve as infection courts but *B. cinerea* can also directly penetrate healthy plant tissues, by secreting β-glucosidase, pectin methylesterase, polygalacturonases and aspartate proteinase which can degrade host cell walls [6]. Agronomic practices alone cannot prevent the disease in many grape-growing area, so chemical treatments should normally be applied [7]. During postharvest storage, many countries have banned treatments with synthetic fungicides that can have a dangerous impact on human health. As a consequence, the use of sulfur dioxide (SO_2_) generator pads is the most common commercial method to control the grey mold in stored table grapes. This strategy has been relatively successful worldwide because of its efficiency, ease of use, affordable cost, and low health risk when compared with fungicides [8]. The SO_2_ pads contain sodium meta-bilsulfite (Na_2_S_2_O_5_), which reacts with the moisture within the carton of grapes to release SO_2_, that is able to kill and eliminate any actively growing *B. cinerea* spores [9]. However, an excessive dose of SO_2_ can damage table grape, causing early browning of the rachis, berry cracking, fruit injury, and allergies in consumers [8]. Therefore, especially for export purposes, the EU countries proposed 10 ppm as the maximum level of SO_2_, in order to protect consumers and the environment. For these reasons, several alternative strategies have been proposed in the postharvest management of *B. cinerea* rots, including chemical [10], biological [11], and physical means [11,12]. In line with environmental-friendly approaches, ‘organic viticulture’ continues to increase globally. It is defined by the International Federation of Organic Agriculture Movement (IFOAM) as a holistic production management system which promotes and enhances agro-ecosystem health, including biodiversity, biological cycles, and soil biological activity. It emphasizes the use of management practices in preference to the use of off-farm inputs, considering that regional conditions require locally adapted systems [13].

Among different biological approaches, the use of the microbial antagonists like yeasts, fungi, and bacteria is quite promising and gaining popularity. In particular, the simple nutritional requirements, the ability to colonize dry surfaces for long periods of time and to grow rapidly on inexpensive substrates in bioreactors [14] and inability to produce allergenic spores or mycotoxins [15,16] are few of the features making yeasts as good Biological Control Agents (BCAs). As recently reviewed [17,18], non-*Saccharomyces* yeasts have been explored as biological control agents against different spoilage fungi, including *B. cinerea*. Different products based on yeasts reached advanced stages of development and commercialization such as Aspire^TM^ (based on *Candida oleophila* Kaisha & Iizuka), Yieldplus^TM^ (based on *Cryptococcus albidus* Skinner) and Shemer^TM^, (based on *Metschnikowia pulcherrima* Pitt & Mill). However, the widespread use of postharvest biocontrol products is still limited, due to their inconsistent performance under commercial conditions as well as the limited market and small size companies involved in their development and commercialization [19].

The first relevant step in developing BCAs is the isolation in their natural environments, in which they compete with plant pathogens [20,21]. For this reason, in this study, we aim to isolate different yeasts from table and wine cultivars healthy grapes in Southern Italy and evaluate their efficacy to control *B. cinerea* in both in vitro and in vivo conditions.

In detail, we isolate five yeast strains and characterize these at both a morphological and molecular level. We test their potential as BCA by in vitro assays, both at 4 °C and 25 °C, and test their potential as BCAs in semi-commercial use. Finally, we test their putative mechanism of action by investigating the ability to produce compounds with specific enzymatic activity against the pathogen. Overall, our data will facilitate future applications in organic agriculture, offering a wider repertoire of yeasts to be used as possible Biological Control Agents (BCAs).

## 2. Materials and Methods

### 2.1. Yeast and Pathogen Isolation and Culture Conditions

Yeasts used in this study were isolated from grape juice produced from berries belonging to the Apulian wine varieties “Negroamaro”, “Aleatico”, and “Primitivo” and to new genotypes of table grape, deriving from the breeding program in progress at CREA-Viticulture and Enology of Turi and currently identified with the codes ‘N20/006′and “N10. The main characteristics of the two genotypes are reported in Table S1 (Supplementary Materials). Of note, bunches were sampled at the beginning of October in the experimental vineyard at CREA-VE, (Turi (BA), Southern Italy) which was not subjected to specific treatment against grey mold. Selected bunches were sampled from plants that showed tolerance to grey mold and totally void of any sign of infection. The grapes were aseptically placed in a full-page microperforated-filter blender bag (filter porosity = 63 microns) (Bag Filter^®^—Interscience Bag System^®^, St Nom, France) and manually crushed to obtain 500 mL of grape juice ready for the isolation protocol. Appropriate dilutions of grape juice for each variety were performed before the plating step in YPDA-Cm (Yeast Extract Peptone Dextrose Agar; 1% yeast extract (Thermo Fisher, Kendel, Germany), 2% peptone (VWR Chemicals, Leuven, Belgium), 2% dextrose (VWR Chemicals, Leuven, Belgium), 2% Agar (VWR Chemicals, Leuven, Belgium), amended with 10 mgmL^−1^ of chloramphenicol (Sigma Aldrich, St. Louis, MO, USA). Plates were incubated for 2–3 days at 25 °C. When colonies obtained from each variety resulted larger than 0.25 ± 0.05 mm [22], they were classified in different groups on the basis of their morphology and/or color. A representative colony from each group was selected and streaked on YPDA-Cm, grown for 48 h at 25 °C and then stored at −80 °C in liquid YPD with 30% (*v/v*) of glycerol added. The color and morphology of each isolate were further evaluated on Wallerstein Laboratory (WL) Nutrient Medium (VWR Chemicals, Leuven, Belgium), by using descriptors previously reported [23].

The *B. cinerea* isolate was recovered from diseased table grape berries (cultivar Red Globe) in Apulia (Italy). Firstly, we microscopically evaluated the presence of the typical conidiophore branches of *B. cinerea*, and subsequently we tested its virulence in wounded grape berries. In particular, using a sterile needle, we performed artificial wounds along with the equatorial area of 20 mature berries of the “Red Globe” cultivar, previously disinfected by dipping for 2 min in 2% (*w/v*) of sodium hypochlorite (NaClO) solution, they were then rinsed with sterilized water and then air dried. The artificial wound was inoculated with 20 µL drop of 1 × 10^5^ conidia mL^−1^ of *B. cinerea*. Berries were placed on a wet paper, which was inserted into boxes that were coated with a plastic film. After 5 days of incubation at 25 °C, we visually evaluated the ability of our *B. cinerea* isolate to develop the common symptoms of the disease. The isolate was stored at 25 °C on petri dishes containing Potato Dextrose Agar (PDA) (VWR Chemicals, Leuven, Belgium).

### 2.2. Molecular Identifications

Yeast DNA was extracted through the Fast DNA^®^ SPIN kit for Soil (MP Biomedicals, LLC, Solon, OH, USA), following the manufacturer’s protocols. Briefly, liquid YPD, containing 48-h-old cells of each yeast strain, were added to a lysing matrix, treated with lysis buffer, and subjected to bead beating in the FastPrep^®^ instrument. DNA was bound to a silica matrix, washed, and eluted in DNase-free water. DNA concentration and purity were assessed spectrophotometrically.

The identification and characterization of the isolates were then verified by the sequencing of 18S and 5.8SrDNA [24]. The NS1⁄ITS2 primer pair with the following sequences was used to amplify the ITS1 region of the 18S rDNA:NS1 5′-GTAGTCATATGCTTGTCTC-3′ and ITS2 5′-GCTGCGTTCTTCATCGATGC-3′ [24]. The ITS1/ITS4 primer pair with the following sequences was used to amplify the 5.8S: ITS1 5′-TCCGTAGGTGAACCTGCGG-3′ and ITS4 5′-TCCTCCGCTTATTGATATGC-3′ [25,26]. PCR was performed as follows: initial denaturation at 95 °C for 5 min, repeated denaturation at 95 °C for 1 min (annealing temperature, 60 °C for 30 s), and elongation at 72 °C for 1 min for 30 cycles. The final elongation lasted for 5 min.

Amplified sequences, after an agarose (1%) gel electrophoretic run phase (orange G marker), were purified using QIA quick PCR purification kit (Qiagen, Hilden, Germany) or by gel band purification, following the manufacturer’s protocols, by GFX PCR DNA and gel band purification kit provided by GE Healthcare (www.gelifescieces.com/illustra, 22 February 2021). After purification, each sample was sequenced by using the Sanger sequencing method following the manufacturer’s protocol of Genome Lab Dye Terminator Cycle Sequencing with the Quick start Kit (www.beckmancoulter.com, 22 February 2021). Subsequently, yeast isolates were identified and classified at the molecular level by using sequencing approaches. We sequenced the internal transcribed spacer (ITS) region both of the 18S rDNA and the 5.8S-ITS region gene of rDNA [27] and BLAST searched against the RNA/ITS Database available at NCBI public repository in order to deeply identify genus and species of the isolates (Table S2 and supplementary sequencing data in Supplementary Materials).

A sequences BLAST was performed using the National Center for Bio-technology Information database, (http://www.ncbi.nlm.nih.gov, 22 February 2021), by searching for significant alignments in the rDNA/ITS database.

### 2.3. In vitro Assay

The yeast strains were grown in flasks containing liquid YPD for 48 h at 25 °C and stored at 4 °C, ready for their use in in vitro assays. A cellophane agar layer technique (CALt) [28] was used in order to evaluate the ability of yeasts to reduce the in vitro mycelial growth of *B. cinerea*. Disc of 90 mm diameter were placed individually between two rectangular sheets of blotting paper, wrapped with aluminum foil, and autoclaved at 121 °C for 20′. A sterilized disc of cellophane was laid aseptically over the solidified YPDA medium in the culture plates. The plates were laid out overnight to allow the excess moisture to evaporate. A volume of 50 µL (at the concentrations of 1.5 x 10^7^ CFUmL^−1^) of each yeast was uniformly distributed on the cellophane disc. A volume of 50 µL of sterilized water was used as the control on a different plate. For each yeast and control, five replicates were produced. After 2 days of incubation at 25 °C, the cellophane discs with and without yeast were removed from the plates. An agar block of 9 mm diameter, cut from the actively growing margins of *B. cinerea*, was placed at the center of all plates and incubated at 25 °C. The radial growth of the colony on 2nd, 4th, 6th, 8th, and 10th days of incubation was measured, and daily mycelial growth was calculated with the following formula.

(1) $$\frac{\sum_{i=0}^{n}\left(y_{n+1}-y_{n}\right)}{N}$$

where:

*y_n_* = the diameter of the mycelium of *B. cinerea* at the time *n*

*y_n_*_+1_ = the diameter of the mycelium of *B. cinerea* at the time *n* + 1

*N* = total number of days considered in the experiment

A modified dual culture method was used to specifically evaluate the efficacy of volatile organic compounds (VOCs) from yeast against *B. cinerea* [29]. Aliquots of 20 μL of yeast suspensions (1.5 × 10^7^ CFUmL^−1^) were seeded on plates containing YPD agar and incubated at 25 °C for 48 h. A mycelial disc (9 mm square plugs) of *B. cinerea* was placed on plates containing YPD agar. The plates with *B. cinerea* disc were individually covered face to face with dishes containing 48-h-old yeast isolates, in order to create a microenvironment suitable for the diffusion of volatile compounds eventually produced by tested yeast isolates. The control was prepared by using YPD agar plates without yeasts. The two plates were firmly wrapped together with Parafilm to prevent air leakage and incubated at 25 °C. Radial growth of *B. cinerea* was calculated as previously described. In both experiments for each yeast strain and control, five plates (replicates) were used and the average daily mycelial growth between replicates was calculated.

### 2.4. Extracellular Lytic Enzymes Activity

The ability of selected yeast to produce lytic enzymes (such as chitinase, β-1,3-glucanase, pectinase, protease, and lipase) was assessed by growing selected isolates on specific growth media for 7 days at 25 °C and 30 days at 0 °C. In particular: (i) chitinase activity was evaluated on solid media containing colloidal chitin, prepared with the second method reported by Roberts and Selitrennikoff (1988) [30] and mineral salts reported by Souza et al., (2009) [31]. Extracellular chitinase activity was detected by the presence of a clear zone around the inoculum zone; (ii) β-1,3-glucanase activity was evaluated on solid medium (pH = 7), containing 5.0 g L^−1^ glucan (Tokyo Chemical Industry, Tokyo, Japan), 6.7 g L^−1^ Yeast Nitrogen Base (YNB) (VWR Chemicals, Solon, OH, USA) and 15.0 g L^−1^ Agar. After the incubation period, the plates were stained with Congo Red (Sigma Aldrich, St. Louis, MO, USA) (0.6 g L^−1^) and left at room temperature for 90 min. The stain not adsorbed was decanted and the hydrolysis of glucan was evaluated by the presence of a yellow-orange zone around the colonies [32]; (iii) pectinase activity was evaluated on solid medium (pH = 7) containing citrus pectin (Thermo Fisher, Kendel, Germany) 10 g L^−1^, YNB 6.7 g L^−1^ and Agar 15 g L^−1^. After incubation at 25 °C and 0 °C, plates were flooded with hexadecyltrimethylammonium bromide (Alfa Aesar GmbH & Co., Karlsruhe, Germany) (10 g L^−1^) and the presence of a clear zone around the colony indicated the degradation of pectines [33]; (iv) protease activity was evaluated on YPDA amended with casein (Merk KGaA, Dermstadt, Germany) 20 g L^−1^. The presence of a clear zone around the colony indicated the ability of isolates to degrade the casein [34]; (v) lipase activity was evaluated on tributyrin agar medium reported by Buzzini and Martini, (2002) [33]. After the incubation period, a clear zone around the colony was detected. For each yeast isolate, 10 μL of a culture suspension (10^7^ CFU mL^−1^) were spotted on three plates of each growth media. For each yeast strain and growth media, the ability to hydrolyze the specific tested compound (presence of a clear or colored zone around the colony) was reported as a positive response (+).

### 2.5. Bioassays Antagonistic Activity

All yeast isolates used in the in vitro assays were evaluated for their ability to control *Botrytis* bunch rot, both in wounded berries and in semi-commercial experimental trials.

Mature berries from healthy bunches of the “Red Globe” cultivar were surface disinfected by dipping for 2 min in 2% (*w/v*) of sodium hypochlorite (NaClO) solution, rinsed with sterilized water, and then air dried. Artificial wounds were performed using a sterile needle along with the berry equatorial areas. We evaluated the ability of yeast to control Botrytis bunch rot at two different concentrations 1.5 × 10^7^ and 1.5 × 10^4^ CFUmL^−1^. In both experiments, each wound was inoculated with a 20 µL drop of yeast suspensions, and 21 grape berries (three replicates each consisting of seven grape berries) for each yeast isolate were placed on plastic boxes. Twenty-one berries inoculated with 20 µL of sterilized water were used as the control. To create a humid environment, a wet paper was placed on the bottom of the boxes that was coated with a plastic film. The trays were incubated at 25 °C for 48 h. After this period, each wound was inoculated with a 20 µL drop of 1 × 10^5^ conidiamL^−1^ of *B. cinerea*. A complete experimental design with three repetition for each treatment was performed. After 5 days of incubation at 25 °C, the disease severity (DS) was evaluated by using an empirical 0-to-4 rating scale (0 = no visible symptoms; 1 = sporulation covering 5–10% of the wound surface; 2 = sporulation covering 10–25% of the wound surface; 3 = sporulation covering 25–50% of the wound surface; 4 = sporulation covering more than 50% of the wound surface). The average of the disease severity was calculated for each plastic box by using the Townsend and Heuberger’s formula [35]. Furthermore, on the basis of the empirical scale, the disease incidence (calculated as the percentage ratio between the number of infected berries and the total number of berries evaluated) and the Mckinney’s Index [36] were calculated.

For the experimental trial in semi-commercial conditions, twenty bunches of the “Red Globe” cultivar were harvested in an experimental vineyard of CREA-VE, located in Rutigliano (BA), southern Italy. All the berries with pedicels were detached from these bunches and divided on the basis of their content in Total Solid Soluble by flotation in different salt solutions. A total of 420 berries were taken from the salt solution corresponding to a soluble solid content between 16% and 17%. The surface of berries was disinfected by dipping for 2 min in 2% (*w/v*) of sodium hypochlorite (NaClO) solution, rinsed with sterilized water, and then air dried. Three hundred of them were divided into five groups (60 berries for each group), and each was individually inoculated with the five yeast strains by dipping the berries for 2 min in 5 L tanks containing a yeasts suspension at the concentration of 1.5 × 10^4^ CFUmL^−1^. The remaining 120 berries were put for 2 min in sterile water and then divided into two groups used as the control: the first group was stored in three boxes with SO_2_ generator pads (positive control), while the second group was stored in three boxes without any further treatment (negative control). After air drying, berries were putted on 21 plastic boxes (three boxes for each treatment), covered with a plastic film, and then placed in a cold refrigerator at 0 °C for 48 h. After this period, berries from each treatment were artificially inoculated by dipping for 2 min in 5 L tanks containing a conidial suspension (1 × 10^5^ conidiamL^−1^) of *B. cinerea,* replaced in their boxes, air dried, and then placed in cold storage for 30 days. At the end of the experiment, the following parameters in each treatment were evaluated: the percentage reduction in berry weight, the incidence of ‘slip skin’ (ISS), browning of pedicel severity (BPS), and sporulation in the pedicel severity (SPS). Reduction in berry weight was evaluated as the *delta* percentage between the berry weight at the beginning of the experiment and the berry weight at the end of the experiment. The ISS was evaluated as the ratio between the number of berries that showed ‘slip skin’ phenomena and the total number of berries. The BPS was evaluated by using an empirical 0-to-4 rating scale (0 = pedicel without visible browning, 1 = surface browned just visible to 25% of the pedicel surface, 2 = 26–50% of pedicel surface affected by browning, 3 = 51–75% of pedicel surface affected by browning, 4 = more than 75% of pedicel surface affected by browning). The SPS was evaluated by using an empirical 0-to-4 rating scale (0 = uninfected pedicel, 1 = surface mycelia just visible to 25% of the pedicel surface, 2 = 26–50% of the pedicel surface covered with mycelia, 3 = 51–75% of the pedicel surface covered with mycelia, 4 = more than 75% of the pedicel surface covered with mycelia). The average of both browning and sporulation severity was calculated for each plastic box by using the Towsend and Heunberger’s formula [35]. Furthermore, regarding the sporulation of the pathogen in the pedicel, their incidence (SPI) (calculated as the percentage ratio between the number of infected pedicels and the total number of pedicels evaluated) and the relative McKinney’s index [36] were also evaluated.

### 2.6. Statistical Analysis

Data were analyzed using the R package software (v. 3.4.1). The normality and homoscedasticity of variances were determined through the Shapiro-Wilk’s test and Levene’s test, respectively. When normal distribution and homoscedasticity of variances were verified, we performed the analysis of variance using the two-way ANOVA test followed by the post-hoc Tukey test (*p* < 0.05). Otherwise, if no normal distribution and heteroscedasticity variances were assessed, the analysis of variance was performed by using the non-parametric Kruskal–Wallis test followed by a post-hoc Dunn’s test (*p* < 0.05).

## 3. Results

### 3.1. Yeast Isolates and Molecular Identification

A total of five isolates were collected from the experimental vineyard in the south of Italy: two isolates, coded N10 and N20/006 (Table S1), were isolated from new table grape crossbreeds (Turi, Bari, Italy), Ale4 and Ale5 were isolated from Aleatico grapes, and Pr7 was isolated from Primitivo variety. Of note, all the isolates belonged to grapes showing good levels of resilience against fungi effects.

The color and morphology of each isolate were analyzed on WL semisolid plates, by using the morphological traits proposed by Pallmann et al., 2001 [23] (Figure S1, Table S2). Overall data revealed that the five isolates belonged to three genera: *Metschnikowia*, *Lachancea*, and *Hanseniaspora,* and, by combining sequencing both of 18S and 5.8S rDNA, this allowed us to assign each isolate to a species: indeed, the three isolates Ale4, N20/007, and Pr7 matched with *M. pulcherrima* with a percentage of sequence identity >97%, we therefore also performed multi-alignment analysis among *M. pulcherrima* sequences and data showed few differences among these three isolates sequences (Supplementary Materials). These findings agree with previously reported data assessing the high diffusion of this yeast species in the Southern Italy area [37]. The other two isolates N10 and Ale5 respectively matched with *L. thermotolerans* and *Hanseniaspora uvarum* (Niehaus) Shehata, Mrak & Phaff*,* with almost 100% identities (Table S2).

### 3.2. Extracellular Lytic Enzymes Activity

To further characterize the five isolates and get clues on their putative mode of action, specific enzymatic activity was investigated. As reported in Table 1, the three *M. pulcherrimma* isolates (N20/006, Ale4 and Pr7) showed different extracellular enzyme activity, thus confirming that they are different strains. In particular, both at mesophilic (25 °C) and psychrophilic (0 °C) temperatures, although all the three isolates were able to hydrolyze tributyrin (lipase activity), *M. pulcherrima* N20/006 showed also pectinase and protease activity, while *M. pulcherrima* Ale4 showed β-1,3-glucanase and pectinase activity. The *L. thermotolerans* N10 was able to hydrolyze both pectines and tributyrin; finally, the *H. uvarum* Ale5 showed β-1,3-glucanase, protease, and lipase activity. None of the yeast isolates tested were able to hydrolyze the chitin.

### 3.3. In Vitro Assay

**Figure 1:**
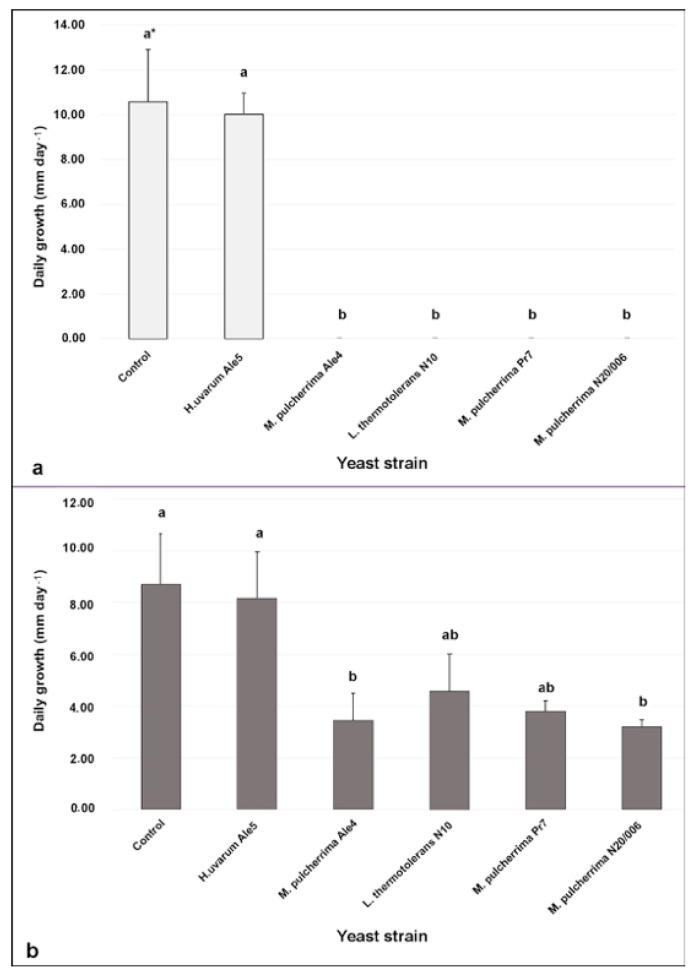
In vitro assays (**a**) cellophane agar layer technique (white bars) (CALt); (**b**) antagonistic activity of volatile organic compounds (VOCs) (grey bars). In vitro antagonistic activity of yeast strains refer to mycelial daily growth of Botrytis cinerea. Data are presented as the mean of five replicates with standard deviation (vertical bars). * Columns labeled by different letters are significantly different according to the Dunn’s test (*p* < 0.05).

The antagonistic role of the five isolates were first evaluated “in vitro”. We tested the ability of yeast isolates to reduce the mycelial daily growth (mmday^−1^) of *B. cinerea* at five time points using two different approaches to investigate the mode of action of each isolate, CALt to investigate the ability to produce antagonistic compound diffusible into the media and VOCs to search for volatile compound with anti-botritic activity (Figure 1). The daily growth reduction was calculated as the percentage reduction of the daily mycelial growth of *B. cinerea* obtained in the presence of the antagonistic yeast isolate compared to the control. We calculated the mycelial daily growth on the basis of the average of the longitudinal and orthogonal diameters of *B. cinerea* colonies, measured every 48 h from the beginning of the experiments until the fungus in the control occupied all the space available in the plates. Data collected from both the in vitro assays were statistically confirmed. On the basis of the results of the Shapiro–Wilk and Levene’s tests, we did not find a normal distribution (*p* < 0.0001, in both assays) and homoscedasity of variance (*p* = 0.051 and 0.22, respectively). Therefore, we performed the analysis of variance using the non-parametric Kuskal–Wallis test and we identified significant differences by the non-parametric Dunn’s test. Results showed that, by using CALt, all the yeast isolates, except for strain *H. uvarum* Ale5, completely inhibited the mycelial growth of *B. cinerea* (Figure 1a).

Similarly, the five yeast isolates showed a different ability to reduce the mycelial growth of *B. cinerea* by VOCs (Figure 1b). In detail, *M. pulcherrima* strains Ale4 and N20/006 significantly reduced the daily growth of the fungus by 60.4 and 61.2%, respectively, while *L. thermotolerans* strain N10 and *M. pulcherrima* strain Pr7, did not significantly reduce the daily growth of *B. cinerea*. The *H. uvarum* strain Ale5 did not show any effect on the mycelial growth of the fungus *B. cinerea*.

### 3.4. Efficacy of the Yeast Cells in Controlling Botrytis Bunch Rot in Wounded Berries

**Figure 2:**
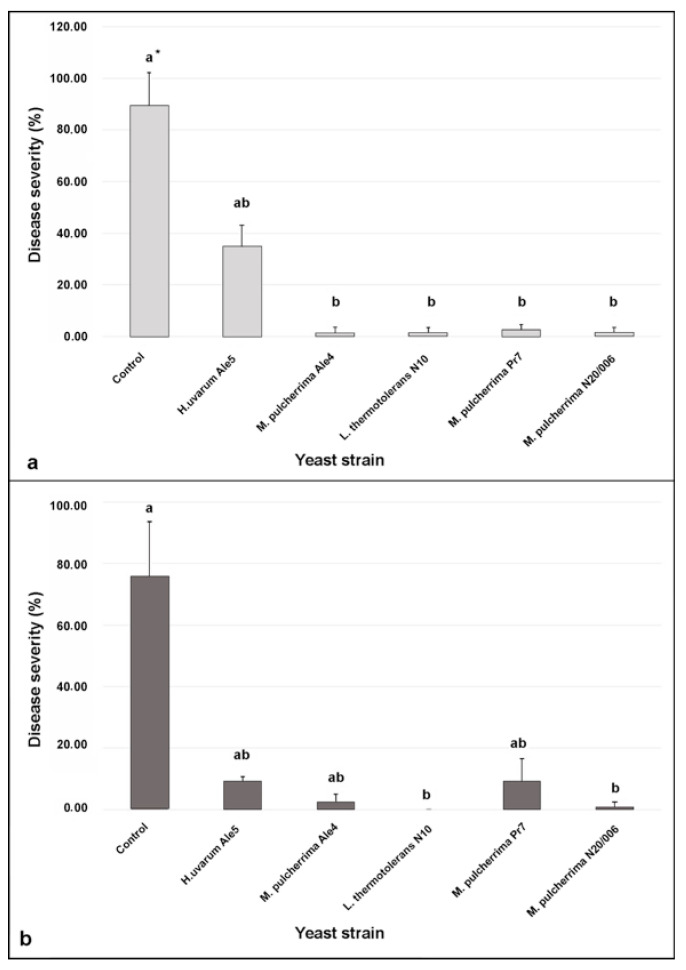
In vivo assay. In vivo antagonistic activity of yeast strains in inhibiting gray mold decay on wounded grape berries at two concentrations: (**a**) report effects of yeasts at 1.5 × 10^7^ CFU mL^−1^ (white columns) and (**b**) report effects of yeasts at 1.5 × 10^4^ CFU mL^−1^ (grey columns). Effects of yeasts refer to the disease severity caused by *Botrytis cinerea* 5 days after incubation at 25 °C. Data are presented as the mean of three replicates with standard deviation (vertical bars). * Columns within the same assay labeled by different letters are significantly different according to the Dunn’s test (*p* < 0.05).

The obtained results revealed that four out of five isolated were good candidates for in vivo assay. Nevertheless, we decided to use all the isolates in the in vivo assays, since the effectiveness of in vitro and in vivo experiments can be largely different mostly related to different mechanisms and interactions between yeasts and *B. cinerea* [38,39]. For this reason, in vivo assays are complementary and essential to confirm the BCA effect. In the in vivo experiments we evaluated the ability of yeast isolates to reduce the disease severity (%), the disease incidence (%), and the McKinney’s Index (%) [36] in wounded berries, artificially inoculated with two different conidia suspensions (1.5 × 10^7^ CFUmL^−1^ and 1.5 × 10^4^ CFUmL^−1^) of *B. cinerea* (Figure 2 and Table S3). Based on the results of Shapiro-Wilk and Levene’s tests, data collected did not show normal distribution (*p* < 0.001, in both experiments) and homoscedacity of variance (*p* = 0.47 and 0.17, respectively); therefore, we performed the analysis of variance by using the non-parametric Kuskal–Wallis test and we identified significant differences by using the non-parametric Dunn’s test. In detail, when the BCAs were applied at the concentration of 1.5 × 10^7^ CFUmL^−1^, DS values of gray mold decay were significantly lower (χ^2^ = 13.21, df = 5, *p* = 0.022) in grape berries treated with the three isolates of *M. pulcherrima* and *L. thermotolerans* strain N10 with respect to the control, likewise to that observed on berries treated with *H. uvarum* strain Ale5. Notably, the *L. thermotolerans* strain N10 and the *M. pulcherrima* strain N20/006 confirmed their ability to significantly reduce (χ^2^ = 15.08, df = 5, *p* = 0.01) the DS of gray mold decay, also when applied at the concentration of 1.5 × 10^4^ CFUmL^−1^. Similar results were obtained regarding the incidence of disease and the McKinney’s index. In detail, the control in both experiments showed the highest incidence of disease (100%) and the McKinney’s index (12.75% and 11.57%, respectively). All the yeast strains (except *H. uvarum* Ale5), when applied at the concentration of 1.5 × 10^7^ CFUmL^−1^, significantly reduced the appearance of the pathogen (reductions of disease incidence between 90.5% and 95.2% and reduction of the McKinney index between 97.3% and 98.7%). The *L. thermotolerans* strain N10 and *M. pulcherrima* strain N20/006, applied at the concentration of 1.5 × 10^4^ CFUmL^−1^, unlike the others, significantly reduced the appearance of the pathogen compared to the control (reduction of disease incidence between 95.3% and 100% and reduction of McKinney’s index between 98.5% and 100%) (Table S3).

### 3.5. Efficacy of the Yeast Cells in Controlling Botrytis Bunch Rot in Semi-Commercial Conditions

For the statistical analysis of data collected from the assay in semi-commercial conditions, we used a multivariate approach. Indeed, data showed normal distribution and homogeneity of variance, thus we performed the MANOVA test, to evaluate the overall variability in the dataset, followed by ANOVA, to evaluate variability in each individual dependent variable and by the Tuckey’s, to evaluate differences among yeasts in each dependent variable. Multivariate analysis of variance (MANOVA) showed statistically significant differences (*p* < 0.0001) between “treatments” (different yeasts strains and control). The subsequent deepening by one-way analysis of variance (ANOVA) showed that these significant differences concern all the dependent variables considered in the study and in ISS (*p* = 0.0029), BPS (*p* < 0.0001), SPS (*p* < 0.0001), SPI (<0.0001) and McKenney’s index (*p* < 0.0001). We did not find significant differences between treatments regarding the percentage reduction in berry weight (*p* = 0.10029) (Table S4).

**Figure 3:**
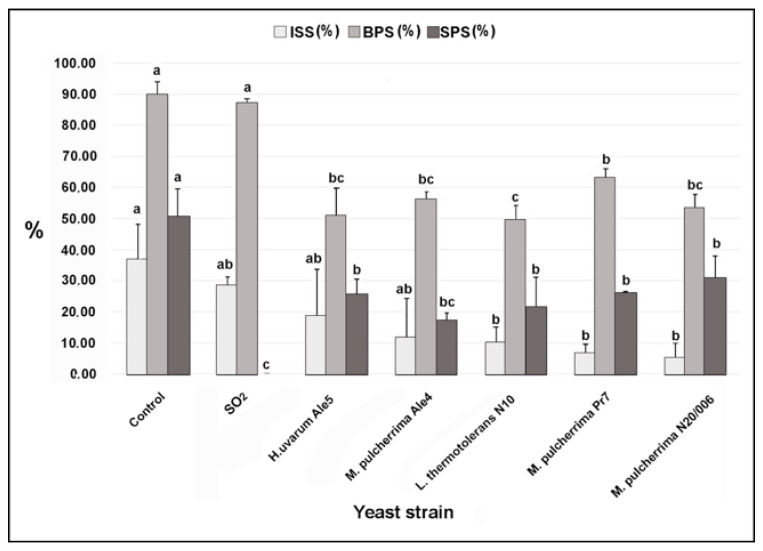
Antagonistic activity of yeast strains in inhibiting gray mold decay on grape berries in semi-commercial conditions. Effects of yeasts refer to the incidence of ‘slip skin’ (ISS), browning of pedicel severity (BPS), and sporulation on pedicel severity (SPS), caused by Botrytis cinerea 30 days after incubation at 0 °C. Data are presented as the mean of three replicates with the standard deviation (vertical bars).

Red Globe berries without treatment showed the highest values both in ISS (36.67%), BPS (89.58%) and SPS (50.42%) (Figure 3). Furthermore, they also showed the highest values of both SPI (90.0%) and McKinney’s Index (2.52%), calculated on the basis of the arbitrary scale used to assess the severity of *Botrytis* sporulation on the pedicel (Table S5). The different tested treatments showed a different ability to reduce these parameters. In detail, *L. thermotolerans* strain N10 and *M. pulcherrima* strain Pr7 and N20/006 showed the highest ability to reduce the ISS compared to the control (72.7%, 81.8%, and 86.4%, respectively). Furthermore, Red Globe berries treated with these yeasts showed significantly lower values than control of both BPS (49.58%, 62.92% and 53.33% respectively) and SPS (21.25%, 25.83% and 30.83%, respectively). *H. uvarum* strain Ale5 and *M. pulcherrima* strain Ale4 showed significantly lower values than the control of both BPS (50.83% and 56.25%, respectively) and SPS (25.42% and 17.08%, respectively). However, these two yeast strains did not significantly reduce the ISS (18.33% and 11.67%, respectively) compared to the control. Treatment with SO_2_ generator pads completely inhibited the SPS, but it was unable to significantly reduce both the ISS (22.7%) and the BPS (2.8%), compared to the control. About the presence of the pathogen on pedicel, only the treatment with the SO2 generator pads was able to completely inhibit both the SPI and the McKenney’s Index (100% in reduction compared to the control) (Supplementary Table S5). Conversely, the tested yeasts isolates did not significantly reduce the SPI compared to the control, but in all cases, the McKinney’s Index was significantly lower than control. In detail, the three *M. pulcherrima* strains were able to reduce the McKinney’s index by 38.3, 48.8 and 95.6%, respectively; the *H. uvarum* strain Ale5 significantly reduced the McKinney’s index by 49.6% and the *L. thermotolerans* strain N10 significantly reduced the McKinney’s index by 57.9%, compared to the control (Table S5).

## 4. Discussion

Postharvest fungal pathogens, such as *B. cinerea*, are considered the main cause of losses of fresh fruit quality during storage, distribution, and consumption. Among the approaches suggested for managing postharvest decay, the use fungicidal treatments is considered unsustainable, because of the emergence of a fungicide-resistance strain of fungal pathogens [40,41,42,43,44] and the dangerous effects on human and environmental health. The use of SO_2_ is, instead, permitted as adjuvant, and it is effective in reducing postharvest fungal pathogens, such as *B. cinerea*. However, alternative more sustainable approaches to SO_2_ are required when considering the hazards for human health and the damages that excessive SO_2_ can cause in stored fresh fruits. In addition, SO_2_ is not allowed as a postharvest treatment for use in organic production [45]. Biological control with microbial antagonists has emerged as a promising alternative to reduce the use of chemical compounds [20,46]. In our study, we isolated five autochthonous yeasts sharing many features as BCAs [14,15,16] against *B. cinerea* and tested them on plate tests and on artificial wounds and semi commercial conditions. We recovered the five isolates from the fructoplane of berries that are representative of the environment that normally is occupied by the pathogen and where the yeasts are able to colonize and survive and compete against antagonists [47].

The recovered isolates, firstly selected on their morphology and/or color, were subsequently identified by sequencing [24]. The identified isolates belong to the non-*Saccharomyces* yeast species, which have been previously isolated from vineyards, spontaneous fermentations [37], and table grape berries [4] in the Apulia region. In particular, the species *M. pulcherrima*, *A. pullulans*, and *H. uvarum* are good candidates as BCAs.

The results of the antagonistic activity in vitro and in vivo assays demonstrated that all the yeast isolates, except for *H. uvarum* strain Ale5, were able to inhibit the mycelial growth of *B. cinerea* at different levels. We hypothesize that the high number of yeasts (4/5) showing BCA effect might be related to the selection bias, since we isolated our yeasts starting from bunches showing tolerance to *B. cinerea* directly on the field. These data is related to the mechanisms of co-evolution of microorganisms with the growing areas and their interaction with the genotype of the host plant, suggesting, in our opinion, that the possibility of selecting co-evolved microorganisms in the same habitats is useful to increase the probability of identifying effective BCAs. Of note, previous data by Qin [48] showed that a proper combination of some *H. uvarum* strains and chemical reagent can provide an effective strategy to reduce postharvest decay of grape berries. Moreover, more recently, *H. uvarum*, isolated from *V. vinifera* ssp. *sylvestris*, has been described as a useful biocontrol agent able to create a protective biofilm [46]. We hypothesize that these contrasting results might be due to the use of different isolated strains and their metabolic characteristics. Indeed, these findings confirmed that the yeast strains isolated have significant differences on the effectiveness against harmful fungi [46]. Moreover, it suggests the importance to further investigate the microbiome diversity to understand how microorganism evolution could influence the resilience of vines and how they adapt to environmental conditions.

The in vitro tests shed light on the different biocontrol mechanism of actions of the yeasts, such as: (i) production of antifungal compounds, (ii) production of VOCs, and (iii) production of cell wall-degrading enzymes. In particular, the ability of the three isolates of *M. pulcherrima* strains Ale4, N20/006, and Pr7 and the *L. thermotolerans* strain N10 to completely inhibited the mycelial growth of *B. cinerea* in the cellophane-agar assays, indicating that they are able to produce fungistatic compounds. These, passing through the pores of cellophane and spreading into the substrate, are able to arrest the growth of *B. cinerea*. This hypothesis is confirmed by the results of an in vitro extracellular lytic enzymes activity assay, in which all the three isolates of *M. pulcherrima* used in this work were able to hydrolyze lipids. Moreover, the *M. pulcherrima* strain Ale4 shown extracellular β-1,3-glucanase and pectinase activity, and the *M. pulcherrima* strain N20/006 showed extracellular pectinase and protease activity. Furthermore, *L. thermotolerans* strain N10 showed both extracellular pectinase and lipase activity. Carbohydrates (such as glucans), proteins, and lipids are the main compounds that constitute the extracellular matrix (ECM) produced by germlings of *B. cinerea* [49,50]. Several possible roles for the ECM produced by *B. cinerea* have been suggested. Among these, there are the involvement in tropism toward the infection site, the prevention of desiccation of conidia, and the provisions of a matrix in which fungal toxins or enzymes required for the infection process could be sequestered [51]. The ability of our isolates to hydrolyze the main compounds that constitute the ECM can explain their ability to control the growth of *B. cinerea* both in in vitro and in vivo assays. Of note, in our work, we revealed that three isolates of *M. pulcherrima* are able to hydrolyze different compounds, thus demonstrating that *M. pulcherrima* not only is one of the most abundant species in the South of Italy, but it is a high versatile species. Different results were found by Parafati et al. (2005) [29]; the authors tested seven isolates of *M. pulcherrima* for their extracellular enzyme activity, including glucanase, chitinase, pectinase, and protease. The seven isolates of *M. pulcherrima* tested by the authors did not shown any extracellular enzyme activity [29].

Our data showed also a significant inhibition effect on the mycelial growth of *B. cinerea* by two isolates of *M. pulcherrima* strain Ale4 and N20/006 through the production of VOCs. These strains were more effective than others belonging to the same species [29,52], while they were less effective than the specific strain of *Starmerella bacillaris* (Kroemer & Krumbholz) Duarte & Fonseca [53]. The effectiveness of our isolates to reduce the mycelial growth of *B. cinerea* by VOCs supports the previous evidence of combined mechanisms of these strains in controlling the growth of fungus [29,52]. However, as in our bioassay the yeast strains were put in direct contact with the pathogen, the main mechanism of action could be related to the release of antifungal compounds and, at a lesser extent, to VOCs. More specific experiments, like those conducted by Parafiti et al. 2015 [29] are necessary in order to confirm if the production of VOCs can represent a main mechanism of action for M. pulcherrima Ale4 and N20/006. Interestingly, in our in vivo experiments, we applied the yeast isolates 48 h before the artificial inoculation with B. cinerea consequently, it is possible that the competition for nutrients and space may be an additional mechanism of action of these yeasts [54,55,56].

The inhibitory effect of our isolates was further proven on wounded grape berries artificially inoculated with *B. cinerea*, in order to confirm the antagonistic ability evaluated in the in vitro experiments. Our in vivo assays showed that the *M. pulcherrima* strains Ale4 and Pr7 were able to control the grey mold of grape berries at the concentration of 1.5 × 10^7^ CFU mL^−1^, while the effectiveness of *M. pulcherrima* strain N20/006 and *L. thermotolerans* strain N10 was independent on their concentration. Strains belonging to the species *M. pulcherrima* [29,57,58,59], as well as to other species, such as *Starmerella bacillaris* [53,60], have already been considered for their high efficacy as biocontrol agents against gray mold of different fruit. However, our isolates were able to control the gray mold at concentrations lower than that previously reported (10^8^ CFU mL^−1^), thus showing a crucial feature for commercial development [61]. The development of a microorganisms for commercial use in biological control involves a series of steps and investments that very often discourage companies to invest in the development of this products. For these reasons, the potential antagonists besides being active against the specific targeted plant pathogen, they must be cost-effective. The ability to be effective also at a low concentration is a key factor to obtain a greater cost-effectiveness of the final product.

Relevant differences have been observed also by comparing the effects of our strains vs. SO_2_ generators pads in semi-commercial conditions on detached grape berries stored at 0 °C, simulating the temperature normally used during cold storage. In particular, all yeast isolates were able to significantly reduce both the incidence of ‘slip skin’ from 50.0% to 86.4% (significantly higher than those obtained by using the SO_2_ generators pads), and the sporulation of the pathogen on the pedicel, from 38.8% to 66.1%, compared to the control. The effectiveness of our yeast isolates could be attributed to the ability of maintaining a high level of viable cells on the fruit surface under storage conditions (low temperature), an important feature for the commercial potential of a postharvest agent. Notably, while other authors have already reported positive effects at cold storage conditions of some strains of *M. pulcherrima* [54,57,62], to our knowledge, this is the first hint regarding *L. thermotolerans*. Of note, *L. thermotolerans* has shown contradictory effect as BCA being inhibitor [63,64] or neutral for the mycelial growth of *A. carbonarius* and *A. niger* [65]. In this study, *L. thermotolerans* strain N10 was effective as BCA against *B. cinerea* both in in vitro and in vivo conditions.

Both *M. pulcherrima* and *L. thermotolerans* have been reported as fermentation partners of *S. cerevisiae*, with inhibiting properties against wine spoilage microorganisms through the production of pulcherrimic acid and killer toxins [66]. The isolation of BCAs could be of interest for their application during winemaking. In particular, several strains of *M. pulcherrima* have a glucosidase activity, a relevant feature able to improve the aromatic profile of the wines [67] and show a high proteolytic activity to serve as a source of nutrients for *Saccharomyces cerevisiae* Meyen [68]. Likewise, selected strains of *L. thermotolerans* have been reported to protect fermenting musts from spoilage microorganisms being producer of lactic acid [68] and improve the release of aroma and color compound through the production of β-D-glucosidase and carbon-sulfur lyase [67] and polymeric pigments [69]. *M. pulcherrima* strains N20/006 and ALE4 and *L. thermotolerans* strain N10 used in this study shown pectinase extracellular activity, that in wine making can be used to obtain positive results in the clarification of white must, to improve the extraction of aroma compounds and to improve the extraction of color and stability of red wine [70]. Further studies in this direction are necessary to clarify the effect of these yeast strains on must fermentation and to determine the nature of the antifungal compounds produced by these yeasts.

In organic viticulture the control of *B. cinerea* infections are usually carried out through canopy management and applications of organic compounds, such as bentonite clays, copper-based formulations, compost teas, or plant extract [7]. These strategies may be variable in effectiveness or have undesirable consequences on the aromatic compounds of some varieties. The yeast we isolated can represent relevant candidates for the technological development of BCA-based agrochemicals that can be used in organic viticulture. Being effective against gray mold both at psychrotrophic and mesophilic temperatures, they could be used both in postharvest and in the vineyard.

## 5. Conclusions

In conclusion, in this work we aimed to isolate yeasts directly from their natural environment (Southern Italy), to be used in stored table grape as BCA control for *B. cinerea* at feasible concentration for commercial use. We isolated different *M. pulcherrima* and one *L. thermotolerans* strains showing inhibitory effects on gray mold decay also at considerably lower concentrations. Future research would help in elucidating the mechanisms of action and in identifying more specific protocols for an ideal postharvest process considering factors such as (i) effectiveness against a wide range of pathogens on different commodities, (ii) adaptability to formulation with a long shelf-life, (iii) possible effect to human health, and (iv) compatibility with commercial processing procedures. Moreover, further studies will need to evaluate and clarify the possible effect of these strains on quality parameters of table grapes such as aroma profile, firmness, taste, total acidity, total antioxidant activity, and color.

## Figures and Tables

**Table 1:** Extracellular lytic enzymes activity of yeast strains. The symbol (+) indicates that the isolate was able to hydrolyze the compound; the symbol (–) indicates that the isolates were not able to hydrolyze the compound.

Yeast Strain	Extracellular Lytic Enzymes Activity									
	Chitinase		β-1,3-glucanase		Pectinase		Protease		Lipase	
	25 °C	0 °C	25 °C	0 °C	25 °C	0 °C	25 °C	0 °C	25 °C	0 °C
*M. pulcherrima* N20/066	−	−	−	−	+	+	+	+	+	+
*M. pulcherrima* Ale4	−	−	+	+	+	+	−	−	+	+
*M. pulcherrima* Pr7	−	−	−	−	−	−	−	−	+	+
*L. thermotolerans* N10	−	−	−	−	+	+	−	−	+	+
*H. uvarum* Ale5	−	−	+	+	+	+	+	+	+	+

## Data Availability

All the sequence data are available in the supplementary materials.

## Supplementary Materials

The following are available online at https://www.mdpi.com/2076-2607/9/2/457/s1, Figure S1: Yeast morphology. Figure S2: In vitro antagonistic tests. Table S1: Agronomic characteristics of the new table grape genotypes. Table S2: Yeast characterization. Table S3: Incidence and McKinney’s Index of grey mold in wounded berries artificial inoculated with two different concentration of yeasts. Table S4: Percentage of reduction in fresh weight of ‘Red globe’ berries treated with different yeast strains and SO_2_ generator pads and stored at 0 °C. Table S5: Incidence and McKinney’s Index of sporulation of *B. cinerea* on pedicel of ‘Red globe’ berries treated with different yeast strains and SO_2_ generator pads and stored at 0 °C.
