# Supplementary material for: Native Vineyard Non-Saccharomyces Yeasts Used for Biological Control of Botrytis cinerea in Stored Table Grape
Source: Microorganisms. 2021 Feb 22;9(2):457. doi: 10.3390/microorganisms9020457 (PMC7926336; doi:10.3390/microorganisms9020457)

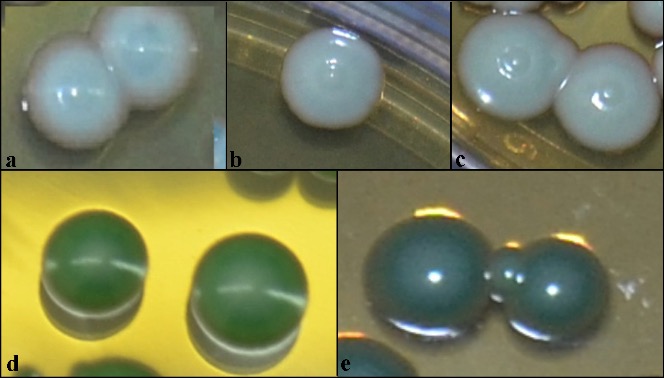


**Figure S1. Yeast morphology.** Color and morphology of each isolate on WL semisolid plates are reported accordingly to Cavazza et al., 1992 and Pallmann et al., 2001. **a** *M.pulcherrima*. N20/006; **b**. *M. pulcherrima* Ale4; **c**. *M. pulcherrima* Pr7; **d**. *L.thermotolerans* N10; **e**. *H. uvarum* Ale5.


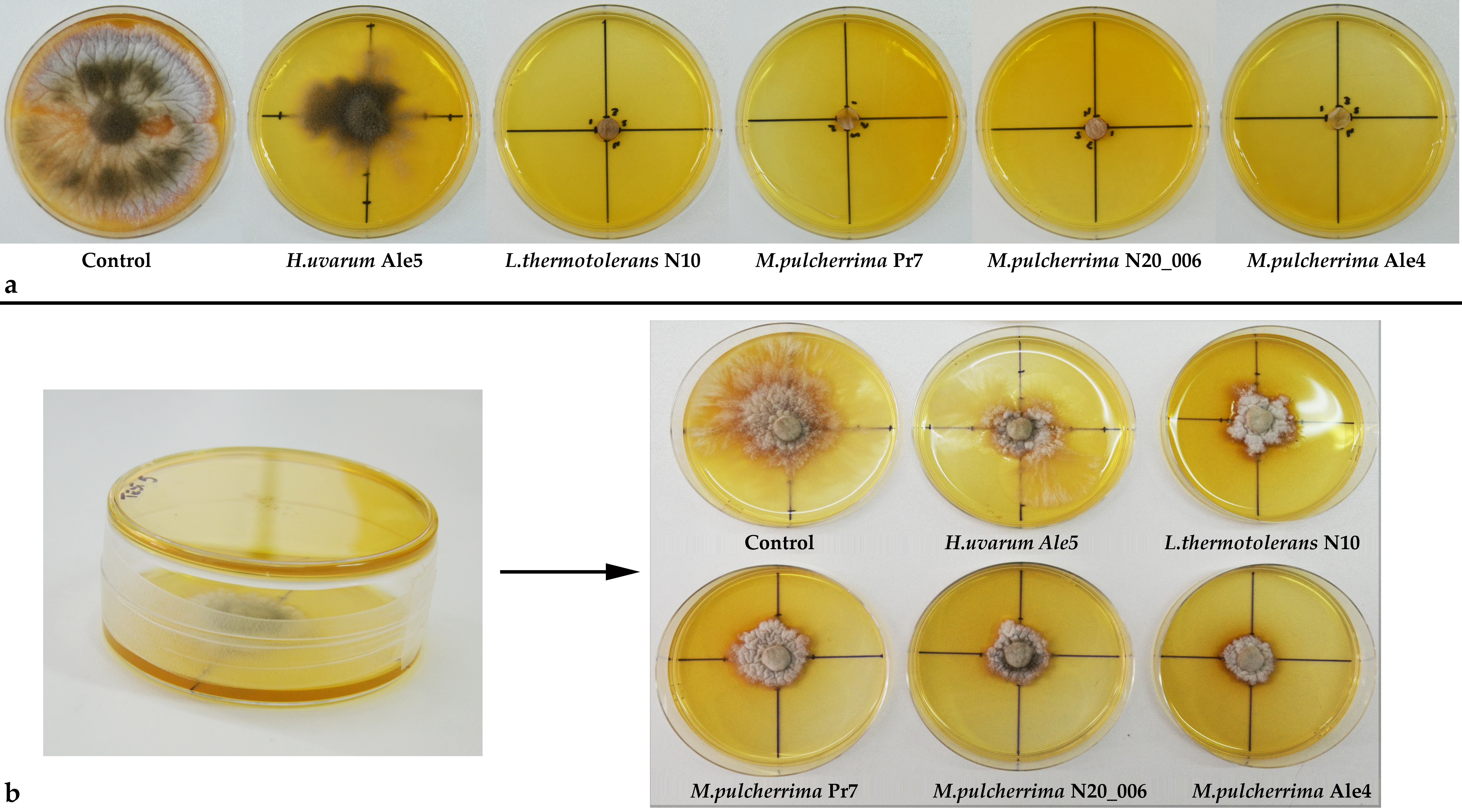


**Figure S2: In vitro antagonistic tests. a.** Cellophane agar *in vitro* antagonistic test**.** Growth of *B. cinerea* in YPDA previously covered with cellophane discs containing the different yeasts strain. **b.** *in vitro* antagonistic test to assess the production of VOCs. Growth of *B. cinerea* in YPDA covered with plates of YPDA with the different yeast strains.

| Genotype code | Berry | | | | | Bunch | |
| --- | --- | --- | --- | --- | --- | --- | --- |
|  | Weight (g)^1^ | Length (mm)^2^ | Color | Formation of seeds^3^ | Weight (g)^4^ | | Lenght (mm)^5^ |
| N20/006 | 4.9 | 17.4 | Red | 1 | 669.0 | | 430.0 |
| N10 | 4.2 | 21.1 | Blu-black | 1 | 322.5 | | 245.0 |

**Table S1. Agronomic characteristics of the new table grape genotypes.**^1^Mean value of 30 typical berries of 5 bunches; ^2^Mean value of 30 non deformed berries taken from the middle part of 10 bunches; ^3^Examination of seeds formation in 30 berries taken from the middle part of 10 bunches. 1=no formation of seeds; 2=seeds with soft seed coat; 3=seeds fully developed; ^4^Mean value of the largest bunch of 10 shoots; ^5^Mean value of the largest bunches of 10 shoots.

|  |  | Colony Morphology | | 18S sequencing match | | | 5.8S sequencing match | | |
| --- | --- | --- | --- | --- | --- | --- | --- | --- | --- |
| Isolate code | Source (*V.vinifera* L.) | Color (front view) | Topography | Closest related type strain (GenBank) | E-value | Identity% | Closest related type strain (GenBank) | E-value | Identity% |
| Strain Ale 4 | Aleativo cv. | Slightly blue, a hint of red-brown in the center | Convex, surface: glossy, radial pattern streak | Uncertain | / | / | M.Pulcherrima (NR_164379.1) | 4e-165 | 97.63 |
| Strain Ale 5 | Aleatico cv. | Intensive green in the center | Flat, surface: smooth, opaque. Consistency of butter. | *H.uvarum* (NG_063250.1) | 0 | 97 | Uncertain | / | / |
| Strain N10 | New table grape crossing | Dark green, cream at the periphery | Convex, surface: smooth, glossy, opaque | L.thermotolerans (NG_061071.1) | 0 | 99.63 | L.thermotolerans (NR_11334.1) | 0 | 98.89 |
| Strain N20/006 | New table grape crossing | Blue green, transparency at the periphery | Convex, surface: smooth, glossy | Uncertain | / | / | M.Pulcherrima (NR_164379.1) | 2e-162 | 97.87 |
| Strain Pr7 | Primitivo cv. | Blue green, transparency at the periphery | Convex, surface: smooth, glossy | M.pulcherrima (NG_065490.1) | 0 | 99.81 | M.Pulcherrima (NG_065490.1) | 2e-172 | 97.8 |

**Table S2. Yeast characterization**. Code and source of each isolate are indicated, column C and D refer to the description of different yeast isolates on WL medium basing on the descriptors proposed by Pullmann et al., 2001.; columns E-H report BLAST main results. Sequence analysis have been reported accordingly GenBank output.

| Yeast strain | yeasts at 1.5 x 10^7^CFU mL^-1^ | | yeasts at 1.5 x 10^4^CFU mL^-1^ | |
| --- | --- | --- | --- | --- |
|  | **Incidence (%)** | **McKinney’s Index (%)** | **Incidence (%)** | **McKinney’s Index (%)** |
| Control | 100±0a | 12.75±1.50a | 100±0a | 11.57±1.58a |
| *H. uvarum*  Ale5 | 100±0a | 6.12±0.83ab | 100±0a | 7.25±0.84ab |
| *M. pulcherrima* Ale4 | 9.52±13.47b | 0.34±0.48b | 14.29±16.66ab | 0.51±0.42ab |
| *M. pulcherrima* Pr7 | 4.76±6.73b | 0.34±0.24b | 23.80±6.73ab | 1.19±1.34ab |
| *L. thermotolerans* N10 | 4.76±6.73b | 0.17±0.24b | 0.00±0b | 0.00±0b |
| *M. pulcherrima* N20/066 | 4.76 ±6.73b | 0.17±0.24b | 4.73±6.73b | 0.17±0.24b |

**Table S3:** **Incidence and McKinney’s Index of grey mold in wounded berries artificial inoculated with two different concentration of yeasts.** Data are presented as mean of three replicates with standard deviation Values within columns followed by the same letters are not significantly different (Dunn’s test; P<0.05)

| Yeast strain | Reduction in fresh weight (%) |
| --- | --- |
| Control | 0.70±0.58 |
| *M. pulcherrima* N20/006 | 1.78±0.17 |
| *M. pulcherrima* Pr7 | 1.68±0.73 |
| *H. uvarum* Ale5 | 1.11±0.80 |
| *L. thermotolerans*N10 | 0.67±0.33 |
| *M. pulcherrima* Ale4 | 1.61±0.11 |
| *SO_2_* | 0.84±0.44 |

**Table S4:** **Percentage of reduction in fresh weight of 'Red globe' berries treated with different yeast strains and SO_2_ generator pads and stored at 0°C.** Data are presented as mean of three replicates with standard deviation.

| Yeast strain | SPI (%) | McKinney’s Index (%) |
| --- | --- | --- |
| Control | 90.0±4.08 a | 2.52±0.37 a |
| *M. pulcherrima* N20/006 | 90.0±7.07 a | 1.54±0.29 b |
| *M. pulcherrima* Pr7 | 90.0±4.08 a | 1.29±0.03 b |
| *H. uvarum* Ale5 | 81.67±6.23 a | 1.27±0.21 b |
| *L. thermotolerans* N10 | 71.67±6.24 a | 1.06±0.41 b |
| *M. pulcherrima* Ale4 | 66.67 ±6.24 a | 0.11±0.11 b |
| *SO_2_* | 0.00±0 b | 0.00±0 c |

**Table S5:** **Incidence and McKinney’s Index of sporulation of *B. cinerea* on pedicel of 'Red globe' berries treated with different yeast strains and SO_2_ generator pads and stored at 0°C.** Data are presented as mean of three replicates with standard deviation. Values within columns followed by the same letters are not significantly different (Turckey's test; P<0.05)


**Supplementary sequencing Data**

**1. Sequencing of 18S rDNA**

>Strain_Ale5

TCCACTTTGTTGCGTACTGTCTTCTCCAGGTCTTTCCTTCTGGTTCTCATTTGGGGTTNACTCCATTNGTTGATCCACGATTTCTNCTTTGAAAAANTTACCAGTGTTCANAGCANGCGTNTTGCTCGAATATATTAGCATGGAATAATAGAATAGGACGATCGGTTCTATTTTGTTGGTTTCTAGGACCATCGTAATGATTAATAGGGACGGTCNGGGGCATCAGTATTCAGNTGTCAGAGGTGAAATTCTTGGATTNACTGAAGACTAACTACTGCGAAAGCATTTGTCAAGGACGTTTTCATTAATCAAGAACGAAAGTTAGGGGATCGAAGATGATCAGATACCGTCGTAGTCTTAACCATAAACTATGCCGACTAGGGATCGGGCGGTGCCTTTTATTGGCCCACTCGGCACCTTACGAGAAATCAAAGTTTTTGGGTTCTGGGGGGAGTATGGTCGCAAGGCTGAAACTTAAAGGAATTGACGGAAGGGCACCACCAGGAGTGGAGCCTGCGGCTTAATTTGACTCAACACGGGAT

>Strain_N10

CGGTCCGATTTTTTTCGTGTACTGGATCCTNCCGAGCCTTTCCTTCTGGCTAACCTTGGGTCACTTGTGGCCCTTGGCGAACCAGGACTTTTACTTTGAAAAAATTAGAGTGTTCAAAGCAGGCGATTGCTCGAATATATTAGCATGGAATAATAGAATAGGACGTTTGGTTCTATTTTGTTGGTTTCTAGGACCATCGTAATGATTAATAGGGACGGTCGGGGGCATCAGTATTCAATTGTCAGAGGTGAAATTCTTGGATTTATTGAAGACTAACTACTGCGAAAGCATTTGCCAAGGACGTTTTCATTAATCAAGAACGAAAGTTAGGGGATCGAAGATGATCAGATACCGTCGTAGTCTTAACCATAAACTATGCCGACTAGGGATCGGGTGGTGTTTTTTTAATGACCCACTCGGCACCTTACGAGAAATCAAAGTCTTTGGGTTCTGGGGGGAGTATGGTCGCAAGGCTGAAACTTAAAGGAATTGACGGAAGGGCACCACCAGGAGTGGAGCCTGCGGCTTAATTTGACTCAACACGGG

>Strain_Pr7

GTCCACTTCTTTGTGAGTACTTTTTGNGGCGGCCCTTCCATGGCCCCTTACAGGGCCATAGTTACTTTGAGTAAATGAGAGTGTTCAAAGCAGGCAAGCGCTTGAATCTTTTAGCATGGAATAATAAAATAGGACGATGATTCTATTTTGTTGGTTTCTAGGACCATCGTAATGATTAATAGGGACGGTCGGGGGCATTAGTATTCAGTTGTAAGAGGTGAAATTCTTAGATTTTCTGAAGACTAACTACTGCGAAAGCATTTGTCAAGGACGTTTTCATTAATCAAGAACGAAAGTTAGGGGATCGAAGATGATCAGATACCGTCGTAGTCTTAACCATAAACTATGCCGACTAGGGATTGGGCGACGCCTCATGTACATGACGCGCCCAGCACCTTACGAGAAATCAAAGTTTTTGGGTTCTGGGGGGAGTATGGTCGCAAGGCTGAAACTTAAAGGAATTGACGGAAGGGCACCACCAGGAGTGGAGCCTGCGGCTTAATTTGACTCAACACGGGA

**2. Sequencing of 5.8S_Internal Transcribed Spacer Region _ ITS1 and ITS4**

>Strain_Ale4_ITS1 ATATTATTAACAACACATTAGTACACTTTTAGGCACAAACTCTAATATCTTAACCGTCAATAACACAATTAAAAAACTTTCAACAACGGATCTCTTGGTTCTCGCATCGATGAAGAACGCAGCGAATTGCGATACGTAATATGACTTGCAGACGTGAATCATTGAATCTTTGAACGCACATTGCGCCCCGGGGTATTCCCCAGGGCATGCGTGGGTGAGCGATATTTACTCTCAAACCTCCGGTTTGGTCCTGCTTCGGCATAATATCAACGGCGCTAGAATAAGTTTTAGCCCCATTCTTTTTCCTCACCCTCGTAAGACTACCCGCTGAACTTAAGCATATCAATAAGCGGAAGGAATCATATATGAGGGACTAT

>Strain_Ale4_ITS4

TCTTACGAGGGTGAGGAAAAAGAATGGGGCTAAAACTTATTCTAGCGCCGTTGATATTATGCCGAAGCAGGACCAAACCGGAGGTTTGAGAGTAAATATCGCTCACCCACGCATGCCCTGGGGAATACCCCGGGGCGCAATGTGCGTTCAAAGATTCAATGATTCACGTCTGCAAGTCATATTACGTATCGCAATTCGCTGCGTTCTTCATCGATGCGAGAACCAAGAGATCCGTTGTTGAAAGTTTTTTAATTGTGTTATTGACGGTTAAGATTTAGAGTTTGTGCCTAAAAGTGTATAAGTTGTTTTTTTAATGATCCTTCCGCAGGTTCACCTA

>Strain_N10 _ITS1

TATTTAGCATTTTGTGTAGAGCAGCCGGGAAAGTTCAGATTGCCTGCGCTTGATTGCGCGGCCGATGATGCTTTCTGTTAACGACTGTCTCTCTACACACACACTGTGGAGTAATTTATTTTACAACGCTTCTTCTTTGGGCTTTACGGCCCAAGGGTTACAAACACAAACAACTATTGTATTTTAAACATTGTCAATTATTTTTCATTTTAGAAAAAAAATATTTAAAACTTTCAACAACGGATCTCTTGGTTCTCGCATCGATGAAGAACGCAGCGAAATGCGATAAGTATTGTGAATTGCAGATATTCGTGAATCATCGAATCTTTGAACGCACATTGCGCCCTCTGGTATTCCAGGGGGCATGCCTGTTTGAGCGTCATTTCCTTCTCAAACCCTCGGGTTTGGTAGTGAGTGGTACTCTTTCTGGGTTAACTTGAAAATGCTGGCCATCTGGCTGTTGCTGACTGAGGTTTTAGTCCAGTCCGCTGATACTCTGCGTATTAGGTTTTACCAACTCGTAGTGGCGTAGTAGGCGTTTTAAAGGCTTTTACTGAAAGTACAGACAGTCTGGCAAACAGTATTCATAAAGTTTGACCTCAAATCAGGTAGGATCACCCGCTGAACTT

>Strain_N10_ ITS4

TACCTGATTTGAGGTCAAACTTTATGAATACTGTTTGCCAGACTGTCTGTACTTTCAGTAAAAGCCTTTAAAACGCCTACTAACGCCACTACGAGTTGGTAAAACCTAATACGCAGAGTATCAGCGGACTGGACTAAAACCTCAGTCAGCAACAGCCAGATGGCCAGCATTTTCAAGTTAACCCAGAAAGAGTACCACTCACTACCAAACCCGAGGGTTTGAGAAGGAAATGACGCTCAAACAGGCATGCCCCCTGGAATACCAGAGGGCGCAATGTGCGTTCAAAGATTCGATGATTCACGAATATCTGCAATTCACAATACTTATCGCATTTCGCTGCGTTCTTCATCGATGCGAGAACCAAGAGATCCGTTGTTGAAAGTTTTAAATATTTTTTTTCTAAAATGAAAAATAATTGACAATGTTTAAAATACAATAGTTGTTTGTGTTTGTAACCCTTGGGCCGTAAAGCCCAAAGAAGAAGCGTTGTAAAATAAATTACTCCACAGTGTGTGTGTAGAGAGACAGTCGTTAACAGAAAGCATCATCGGCCGCGCAATCAAGCGCAGGCTTCTGAACTTTCCCGGCTGCTCTAACAAAATTCTTTAATGATCCCTCCGCA

>Strain_N20.006_ITS1

TATGATAACACACTTGATACACTTTTAGGCACAAACTCTATAATCTTAACCGTCAATAACACAATTAAAAAACTTTCAACAACGGATCTCTTGGTTCTCGCATCGATGAAGAACGCAGCGAATTGCGATACGTAATATGACTTGCAGACGTGAATCATTGAATCTTTGAACGCACATTGCGCCCCGGGGTATTCCCCAGGGCATGCGTGGGTGAGCGATATTTACTCTCAAACCTCCGGTTTGGTCCTGCTTCGGCATAATATCAACGGCGCTAGAATAAGTTTTAGCCCCATTCTTTTTCCTCACCCTCGTAAGACTACCCGCTGAACTTAAGCATATCAATAAGC

>Strain_N20.006_ ITS4

TACGAGGGTGAGGAAAAAGAATGGGGCTAAAACTTATTCTAGCGCCGTTGATATTATGCCGAAGCAGGACCAAACCGGAGGTTTGAGAGTAAATATCGCTCACCCACGCATGCCCTGGGGAATACCCCGGGGCGCAATGTGCGTTCAAAGATTCAATGATTCACGTCTGCAAGTCATATTACGTATCGCAATTCGCTGCGTTCTTCATCGATGCGAGAACCAAGAGATCCGTTGTTGAAAGTTTTTTAATTGTGTTATTGACGGTTAAGATTTAGAGTTTGTGCCTAAAAGTGTATAAKTGTTTTTTTAATGATCCTTCCGCAGGTTCACCTA

>Strain_PR7 _ ITS1

GGGTTCATACGCTTGACCTTCCTATAGCACGAACTCTAAATACTTAACCGTCAATAACACAATTAAAAAACTTTCAACAACGGATCTCTTGGTTCTCGCATCGATGAAGAACGCAGCGAATTGCGATACGTAATATGACTTGCAGACGTGAATCATTGAATCTTTGAACGCACATTGCGCCCCGGGGTATTCCCCAGGGCATGCGTGGGTGAGCGATATTTACTCTCAAACCTCCGGTTTGGTCCTGCTTCGGCATAATATCAACGGCGCTAGAATAAGTTTTAGCCCCATTCTTTTTCCTCACCCTCGTAAGACTACCCGCTGAACTTAAGCATATCAATAAGCGGAGGAAA

>Strain_Pr7 _ITS4

GGGTTCATACGCTTGACCTTCCTATAGCACGAACTCTAAATACTTAACCGTCAATAACACAATTAAAAAACTTTCAACAACGGATCTCTTGGTTCTCGCATCGATGAAGAACGCAGCGAATTGCGATACGTAATATGACTTGCAGACGTGAATCATTGAATCTTTGAACGCACATTGCGCCCCGGGGTATTCCCCAGGGCATGCGTGGGTGAGCGATATTTACTCTCAAACCTCCGGTTTGGTCCTGCTTCGGCATAATATCAACGGCGCTAGAATAAGTTTTAGCCCCATTCTTTTTCCTCACCCTCGTAAGACTACCCGCTGAACTTAAGCATATCAATAAGCGGAGGAAA

**3**. ***M.pulcherrima sequence analysis***

**3.1 Blast2 alignment**

***M.Pulcherrima*_N20.006**

Summary alignment IS1_vs_ITS4


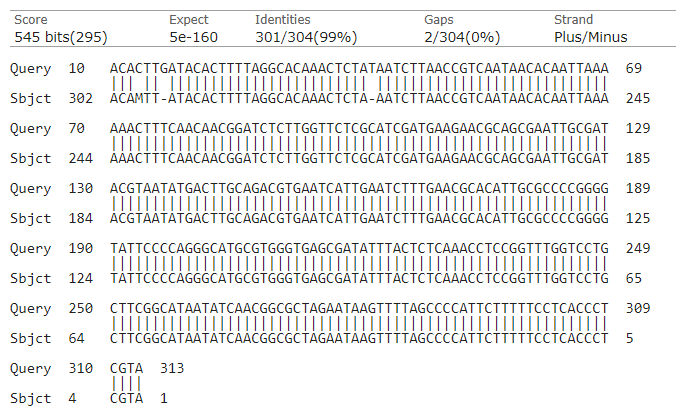


Summary Alignment.SAM

Query_4665 16 Query_4663 10 255 31H6M1D24M1D272M * 0 ACAMTTATACACTTTTAGGCACAAACTCTAAATCTTAACCGTCAATAACACAATTAAAAAACTTTCAACAACGGATCTCTTGGTTCTCGCATCGATGAAGAACGCAGCGAATTGCGATACGTAATATGACTTGCAGACGTGAATCATTGAATCTTTGAACGCACATTGCGCCCCGGGGTATTCCCCAGGGCATGCGTGGGTGAGCGATATTTACTCTCAAACCTCCGGTTTGGTCCTGCTTCGGCATAATATCAACGGCGCTAGAATAAGTTTTAGCCCCATTCTTTTTCCTCACCCTCGTA * AS:i:295 EV:f:5.12854e-160 NM:i:2 PI:f:99.67 BS:f:545.882

>lcl|consensus

TACGAGGGTGAGGAAAAAGAATGGGGCTAAAACTTATTCTAGCGCCGTTGATATTATGCC

GAAGCAGGACCAAACCGGAGGTTTGAGAGTAAATATCGCTCACCCACGCATGCCCTGGGG

AATACCCCGGGGCGCAATGTGCGTTCAAAGATTCAATGATTCACGTCTGCAAGTCATATT

ACGTATCGCAATTCGCTGCGTTCTTCATCGATGCGAGAACCAAGAGATCCGTTGTTGAAA

GTTTTTTAATTGTGTTATTGACGGTTAAGATTATAGAGTTTGTGCCTAAAAGTGTATCAA

GTGT

>lcl|Query_4663

TACGAGGGTGAGGAAAAAGAATGGGGCTAAAACTTATTCTAGCGCCGTTGATATTATGCC

GAAGCAGGACCAAACCGGAGGTTTGAGAGTAAATATCGCTCACCCACGCATGCCCTGGGG

AATACCCCGGGGCGCAATGTGCGTTCAAAGATTCAATGATTCACGTCTGCAAGTCATATT

ACGTATCGCAATTCGCTGCGTTCTTCATCGATGCGAGAACCAAGAGATCCGTTGTTGAAA

GTTTTTTAATTGTGTTATTGACGGTTAAGATTATAGAGTTTGTGCCTAAAAGTGTATCAA

GTGT

>lcl|Query_4665

TACGAGGGTGAGGAAAAAGAATGGGGCTAAAACTTATTCTAGCGCCGTTGATATTATGCC

GAAGCAGGACCAAACCGGAGGTTTGAGAGTAAATATCGCTCACCCACGCATGCCCTGGGG

AATACCCCGGGGCGCAATGTGCGTTCAAAGATTCAATGATTCACGTCTGCAAGTCATATT

ACGTATCGCAATTCGCTGCGTTCTTCATCGATGCGAGAACCAAGAGATCCGTTGTTGAAA

GTTTTTTAATTGTGTTATTGACGGTTAAGATT-TAGAGTTTGTGCCTAAAAGTGTAT-AA

KTGT

***M.Pulcherrima*_Ale4**

Summary alignment IS1_vs_ITS4


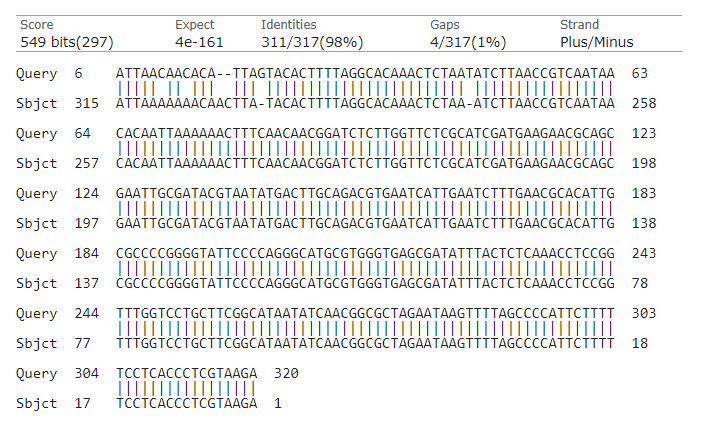


Summary Alignment.SAM

Query_36773 16 Query_36771 6 255 22H12M2I3M1D24M1D274M * 0 0 ATTAAAAAAACAACTTATACACTTTTAGGCACAAACTCTAAATCTTAACCGTCAATAACACAATTAAAAAACTTTCAACAACGGATCTCTTGGTTCTCGCATCGATGAAGAACGCAGCGAATTGCGATACGTAATATGACTTGCAGACGTGAATCATTGAATCTTTGAACGCACATTGCGCCCCGGGGTATTCCCCAGGGCATGCGTGGGTGAGCGATATTTACTCTCAAACCTCCGGTTTGGTCCTGCTTCGGCATAATATCAACGGCGCTAGAATAAGTTTTAGCCCCATTCTTTTTCCTCACCCTCGTAAGA * AS:i:297 EV:f:4.37101e-161 NM:i:4 PI:f:99.36 BS:f:549.575

>lcl|consensus

TCTTACGAGGGTGAGGAAAAAGAATGGGGCTAAAACTTATTCTAGCGCCGTTGATATTAT

GCCGAAGCAGGACCAAACCGGAGGTTTGAGAGTAAATATCGCTCACCCACGCATGCCCTG

GGGAATACCCCGGGGCGCAATGTGCGTTCAAAGATTCAATGATTCACGTCTGCAAGTCAT

ATTACGTATCGCAATTCGCTGCGTTCTTCATCGATGCGAGAACCAAGAGATCCGTTGTTG

AAAGTTTTTTAATTGTGTTATTGACGGTTAAGATATTAGAGTTTGTGCCTAAAAGTGTAC

TAAGTTGTKTTKTTAAT

>lcl|Query_36771

TCTTACGAGGGTGAGGAAAAAGAATGGGGCTAAAACTTATTCTAGCGCCGTTGATATTAT

GCCGAAGCAGGACCAAACCGGAGGTTTGAGAGTAAATATCGCTCACCCACGCATGCCCTG

GGGAATACCCCGGGGCGCAATGTGCGTTCAAAGATTCAATGATTCACGTCTGCAAGTCAT

ATTACGTATCGCAATTCGCTGCGTTCTTCATCGATGCGAGAACCAAGAGATCCGTTGTTG

AAAGTTTTTTAATTGTGTTATTGACGGTTAAGATATTAGAGTTTGTGCCTAAAAGTGTAC

TAA--TGTGTTGTTAAT

>lcl|Query_36773

TCTTACGAGGGTGAGGAAAAAGAATGGGGCTAAAACTTATTCTAGCGCCGTTGATATTAT

GCCGAAGCAGGACCAAACCGGAGGTTTGAGAGTAAATATCGCTCACCCACGCATGCCCTG

GGGAATACCCCGGGGCGCAATGTGCGTTCAAAGATTCAATGATTCACGTCTGCAAGTCAT

ATTACGTATCGCAATTCGCTGCGTTCTTCATCGATGCGAGAACCAAGAGATCCGTTGTTG

AAAGTTTTTTAATTGTGTTATTGACGGTTAAGAT-TTAGAGTTTGTGCCTAAAAGTGTA-

TAAGTTGTTTTTTTAAT

***M.Pulcherrima*_Pr7**

Summary alignment IS1_vs_ITS4


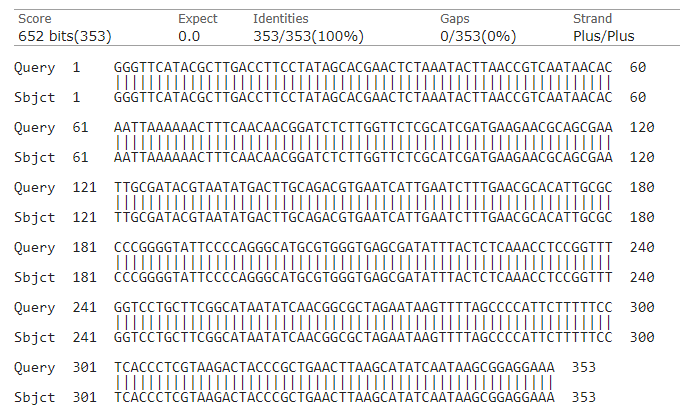


Summary Alignemtn.SAM

Query_27305 0 Query_27303 1 255 353M * 0 0 GGGTTCATACGCTTGACCTTCCTATAGCACGAACTCTAAATACTTAACCGTCAATAACACAATTAAAAAACTTTCAACAACGGATCTCTTGGTTCTCGCATCGATGAAGAACGCAGCGAATTGCGATACGTAATATGACTTGCAGACGTGAATCATTGAATCTTTGAACGCACATTGCGCCCCGGGGTATTCCCCAGGGCATGCGTGGGTGAGCGATATTTACTCTCAAACCTCCGGTTTGGTCCTGCTTCGGCATAATATCAACGGCGCTAGAATAAGTTTTAGCCCCATTCTTTTTCCTCACCCTCGTAAGACTACCCGCTGAACTTAAGCATATCAATAAGCGGAGGAAA * AS:i:353 EV:f:0 NM:i:0 PI:f:100.00 BS:f:652.988

>lcl|consensus

GGGTTCATACGCTTGACCTTCCTATAGCACGAACTCTAAATACTTAACCGTCAATAACAC

AATTAAAAAACTTTCAACAACGGATCTCTTGGTTCTCGCATCGATGAAGAACGCAGCGAA

TTGCGATACGTAATATGACTTGCAGACGTGAATCATTGAATCTTTGAACGCACATTGCGC

CCCGGGGTATTCCCCAGGGCATGCGTGGGTGAGCGATATTTACTCTCAAACCTCCGGTTT

GGTCCTGCTTCGGCATAATATCAACGGCGCTAGAATAAGTTTTAGCCCCATTCTTTTTCC

TCACCCTCGTAAGACTACCCGCTGAACTTAAGCATATCAATAAGCGGAGGAAA

>lcl|Query_27303

GGGTTCATACGCTTGACCTTCCTATAGCACGAACTCTAAATACTTAACCGTCAATAACAC

AATTAAAAAACTTTCAACAACGGATCTCTTGGTTCTCGCATCGATGAAGAACGCAGCGAA

TTGCGATACGTAATATGACTTGCAGACGTGAATCATTGAATCTTTGAACGCACATTGCGC

CCCGGGGTATTCCCCAGGGCATGCGTGGGTGAGCGATATTTACTCTCAAACCTCCGGTTT

GGTCCTGCTTCGGCATAATATCAACGGCGCTAGAATAAGTTTTAGCCCCATTCTTTTTCC

TCACCCTCGTAAGACTACCCGCTGAACTTAAGCATATCAATAAGCGGAGGAAA

>lcl|Query_27305

GGGTTCATACGCTTGACCTTCCTATAGCACGAACTCTAAATACTTAACCGTCAATAACAC

AATTAAAAAACTTTCAACAACGGATCTCTTGGTTCTCGCATCGATGAAGAACGCAGCGAA

TTGCGATACGTAATATGACTTGCAGACGTGAATCATTGAATCTTTGAACGCACATTGCGC

CCCGGGGTATTCCCCAGGGCATGCGTGGGTGAGCGATATTTACTCTCAAACCTCCGGTTT

GGTCCTGCTTCGGCATAATATCAACGGCGCTAGAATAAGTTTTAGCCCCATTCTTTTTCC

TCACCCTCGTAAGACTACCCGCTGAACTTAAGCATATCAATAAGCGGAGGAAA

**4. Multi-alignment *M.pulcherrima_***ClustalW (<https://www.genome.jp/tools-bin/clustalw> )

CLUSTAL 2.1 multiple sequence alignment

N20.006_consensus ------------------------------------TACGAGGGTGAGGAAAAAGAATGG

Ale4_consensus ---------------------------------TCTTACGAGGGTGAGGAAAAAGAATGG

Pr7_consensus GGGTTCATACGCTTGACCTTCCTATAGCACGAACTCTAAATACTTAACCGTCAATAACAC

** * * ** **

N20.006_consensus GGCTAAAACTTATTC--TAGCG----CCGTTGATATTATGCCGAAGCAGGACCAAACCGG

Ale4_consensus GGCTAAAACTTATTC--TAGCG----CCGTTGATATTATGCCGAAGCAGGACCAAACCGG

Pr7_consensus AATTAAAAAACTTTCAACAACGGATCTCTTGGTTCTCGCATCGATGAAGAACGCAGC--G

***** *** * ** * * * * * *** * ** ** * * *

N20.006_consensus AGGTTTGAGAGTAAATATCGCTCACCCACGCATGCCCTGGGGAATACCCCGGGGCGCAAT

Ale4_consensus AGGTTTGAGAGTAAATATCGCTCACCCACGCATGCCCTGGGGAATACCCCGGGGCGCAAT

Pr7_consensus AATTGCGATACGTAATATGACTTGCAGACGTGAATCATTG--AATCTTTGAACGCACATT

* * ** * ***** ** * *** * * * *** ** ** *

N20.006_consensus GTGCGTTCAAAGATTCA--ATGATTCACGTCTGCAAGTCATATTACGTATCGCAATTC--

Ale4_consensus GTGCGTTCAAAGATTCA--ATGATTCACGTCTGCAAGTCATATTACGTATCGCAATTC--

Pr7_consensus GCGCCCCGGGGTATTCCCCAGGGCATGCGTGGGTGAGCGATATTTACTCTCAAACCTCCG

* ** **** * * *** * ** ***** * ** * **

N20.006_consensus GCTGCGTTCTTCATCGATGCGAGAACCAAGAGATCCGTTGTTGAAAGTTTT----TTAAT

Ale4_consensus GCTGCGTTCTTCATCGATGCGAGAACCAAGAGATCCGTTGTTGAAAGTTTT----TTAAT

Pr7_consensus GTTTGGTCCTGCTTCGGCATAATATCAACGG----CGCTAGAATAAGTTTTAGCCCCATT

* * ** ** * *** * * * * * ** * ******* * *

N20.006_consensus TGTGTTATTGACGGTTAAGATTATAGAGTTTGTGCCTAAAAGTGTATCAAGT-GT-----

Ale4_consensus TGTGTTATTGACGGTTAAGATATTAGAGTTTGTGCCTAAAAGTGTACTAAGTTGTKTTKT

Pr7_consensus CTTTTTCCTCACCCTCGTAAGACTACCCGCTGAACTTAA--GCATATCAATAAGCGGAGG

* ** * ** * * ** ** * *** * ** ** *

N20.006_consensus ----

Ale4_consensus TAAT

Pr7_consensus AAA-


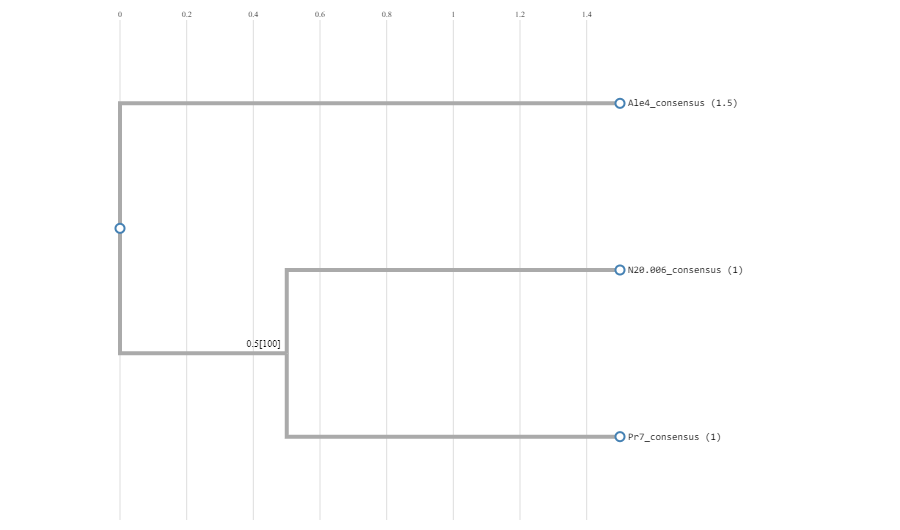

Supplement: Supplementary file 1 [file microorganisms-09-00457-s001.docx]
